# A fern *WUSCHEL-RELATED HOMEOBOX* gene functions in both gametophyte and sporophyte generations

**DOI:** 10.1186/s12870-019-1991-8

**Published:** 2019-10-11

**Authors:** Christopher E. Youngstrom, Lander F. Geadelmann, Erin E. Irish, Chi-Lien Cheng

**Affiliations:** Department of Biology, University of Iowa, 129 E. Jefferson St., Iowa City, Iowa 52242 USA

**Keywords:** Apical cells, *Ceratopteris richardii*, Fern, Gametophyte and sporophyte, Meristem, RNAi, *WUSCHEL-RELATED HOMEOBOX* (*WOX*), Stem cell

## Abstract

**Background:**

Post-embryonic growth of land plants originates from meristems. Genetic networks in meristems maintain the stem cells and direct acquisition of cell fates. WUSCHEL-RELATED HOMEOBOX (WOX) transcription factors involved in meristem networks have only been functionally characterized in two evolutionarily distant taxa, mosses and seed plants. This report characterizes a *WOX* gene in a fern, which is located phylogenetically between the two taxa.

**Results:**

*CrWOXB* transcripts were detected in proliferating tissues, including gametophyte and sporophyte meristems of *Ceratopteris richardii*. In addition, *CrWOXB* is expressed in archegonia but not the antheridia of gametophytes. Suppression of *CrWOXB* expression in wild-type RN3 plants by RNAi produced abnormal morphologies of gametophytes and sporophytes. The gametophytes of RNAi lines produced fewer cells, and fewer female gametes compared to wild-type. In the sporophyte generation, RNAi lines produced fewer leaves, pinnae, roots and lateral roots compared to wild-type sporophytes.

**Conclusions:**

Our results suggest that *CrWOXB* functions to promote cell divisions and organ development in the gametophyte and sporophyte generations, respectively*. CrWOXB* is the first intermediate-clade WOX gene shown to function in both generations in land plants.

**Electronic supplementary material:**

The online version of this article (10.1186/s12870-019-1991-8) contains supplementary material, which is available to authorized users.

## Background

Stem cells are self-renewing pluripotent cells. In vascular plants, they are located in the shoot apical meristem (SAM) and root apical meristem (RAM). Stem cells divide at a low frequency to produce daughter cells that will either maintain the stem cell pool or actively divide and take on new identities to form new organs [1, 2]. The size of a stem cell population varies among different species and is strictly maintained as a part of the meristem [3, 4]. Failure to coordinate multiple inter- and intra-cellular signals disrupts development and results in altered plant body architecture [4, 5]. In addition to hormonal signals, inter-cellular signaling is mediated by small peptide ligands and their cognate receptors. These signals converge to regulate specific transcription factors to achieve a balance among the populations of stem cells, the faster dividing cells, and the differentiating cells of the meristem [6, 7]. In *Arabidopsis thaliana*, the homeobox transcription factor *WUSCHEL* (*WUS*) is a key player in shoot meristem maintenance; *WUS* expression is transcriptionally regulated and the protein acts non-cell-autonomously by moving from the organizing center (OC) to the central zone (CZ) of the SAM to both activate and repress gene transcription in order to maintain meristem cells in a pluripotent state [8].

*WUS* belongs to the family of *WUSCHEL-RELATED HOMEOBOX (WOX)* transcription factors, which are characterized by the presence of a conserved homeodomain [9, 10]. Phylogenetic analyses of land plant *WOX* genes group members into three clades: ancient, intermediate, and modern ( [11] Additional file 1: Figure S1). The progenitor of *WOX* genes existed in the last common ancestor of land plants and green algae and, through successive gene duplication and functional diversification, gave rise to the three clades of *WOX* genes [9, 12]. All land plants that have been examined, non-vascular and vascular alike, possess *WOX* genes of the ancient clade, while the intermediate clade only exists in vascular plants, and the modern clade is found in seed plants and ferns, but has not been found in lycophytes [13, 14]. Based on the presence of two subgroups of the intermediate clade in the lycophytes and sequence relatedness of only one subgroup to the modern clade *WOX* genes, it has been proposed that the intermediate subgroup shared a progenitor with the modern clade [11, 14]. The modern clade, or the *WUS* clade, has experienced further expansion in seed plants as *Picea abies* possesses five and *A. thaliana* possesses eight *WUS* clade members [9, 15], compared to the single member found in the fern, *Ceratopteris richardii* [11]. Modern clade WUS proteins contain, in addition to the canonical homeobox, the WUS box (TL-LFPMILV) [9]. Both conserved domains are required for meristem maintenance in the *A. thaliana* SAM [16]. Maintenance of the SAM [17] and RAM [18, 19] is under the control of *AtWUS* and *AtWOX5*, respectively, and in addition, *AtWOX4* functions in the vascular cambium stem cells [20, 21] where continually dividing cells produce phloem and xylem during secondary growth. All other *AtWOX* genes of the three clades play roles in early embryo development or in organ development, including leaf, root, and floral organs [12, 21–28].

The *WOX* gene family has been widely studied in land plants, including *C. richardii*, but functional studies are limited to seed plants such as *A. thaliana* (e.g. [9, 16],), *Oryza sativa* (e.g. [29],), *P. abies* [30, 31] and moss *Physcomitrella patens* [32]. Five *WOX* genes, *CrWOX13A* and *CrWOX13B* of the ancient clade, *CrWOXA* and *CrWOXB* of the intermediate, and *CrWUL* of the modern clades have been identified in *C. richardii* [11]. RT-PCR results showed that *CrWOX13A* and *CrWOXB* are equally expressed in all tissue examined, including root tip, gametophyte, and young sporophyte; whereas *CrWOXA* was expressed more strongly in the root tip and *CrWUL* in the root tip and gametophyte [11]. In situ hybridization analyses of the latter two genes showed localized expression. *CrWOXA* is expressed in the root apical cell and in the lateral root apical cell. In addition to expression in the vascular bundle of the leaves [33], *CrWUL* mRNA is localized to the cutting edge of the lateral root apical cell which divides asymmetrically, proximal to the main root axis [11]. *CrWOXB* shows a broad expression pattern in the root tip, consistent with the high levels of expression detected by RT-PCR [11].

The unbiased expression of *CrWOX13A* and *CrWOX*B in both gametophyte and sporophyte generations presents an opportunity to understand the ancestral functions of WOX proteins. In the moss *P. patens,* only the ancient clade of *WOX* genes exists, and, in contrast to *A. thaliana* ancient *WOX* genes*, P. patens* ancient *WOX* genes function in both generations [32]. Only two *AtWOX* genes, *AtWOX2* and *AtWOX8,* are expressed in both sporophyte and gametophyte generations [25]; all other *AtWOX* genes seem to function only in the sporophytes [12, 21–28]. Interestingly, in *Nicotiana tabacum*, transcripts of two ancient and one intermediate *WOX* genes are found in both the gametophyte and sporophyte tissues [34].

Sister clade to seed plants, ferns have sporophytic SAMs that are composed of multiple zones resembling that of the seed plants [35, 36]. How these zones are involved in stem cell maintenance and organ initiation is unclear. Moreover, how the fern gametophyte notch meristem is maintained is completely unknown. Thus, *WOX* genes provide an entry point for understanding the meristem of ferns at both the development and the evolution levels. So far, the combination of *WOX* gene family evolution and their developmental functions has only been studied in detail in the moss *P. patens* and angiosperms. Similar investigation in the fern will bridge the gap in our knowledge of meristem evolution. Furthermore, comparison between the gametophyte and the sporophyte meristems within the fern will provide insight into the co-option of gene network between the meristem of the two generations. This understanding can only be fully realized with the expression and functional analyses of all five *WOX* fern genes. Here, we present the completed study of one of the five *WOX* genes found in *C. richardii*, *CrWOXB,* which is expressed in both sporophyte and gametophyte generations [11], to examine its expression in the meristems of sporophyte shoot and gametophyte using sectioned and whole mount in situ hybridization, respectively. The possible function of *CrWOXB* in both generations was examined by RNAi suppression of *CrWOXB* expression in transgenic *C. richardii* plants. These results show that *CrWOXB*, an intermediate-clade *WOX* gene, is expressed in regions of cell proliferation in both the gametophyte and sporophyte. The phenotypes of the RNAi suppression lines were consistent with meristem defects, providing the first demonstration of *WOX* gene function in a fern.

## Results

### *CrWOXB* is expressed in regions of cell division in both the gametophyte and sporophyte generations

**Fig. 1:**
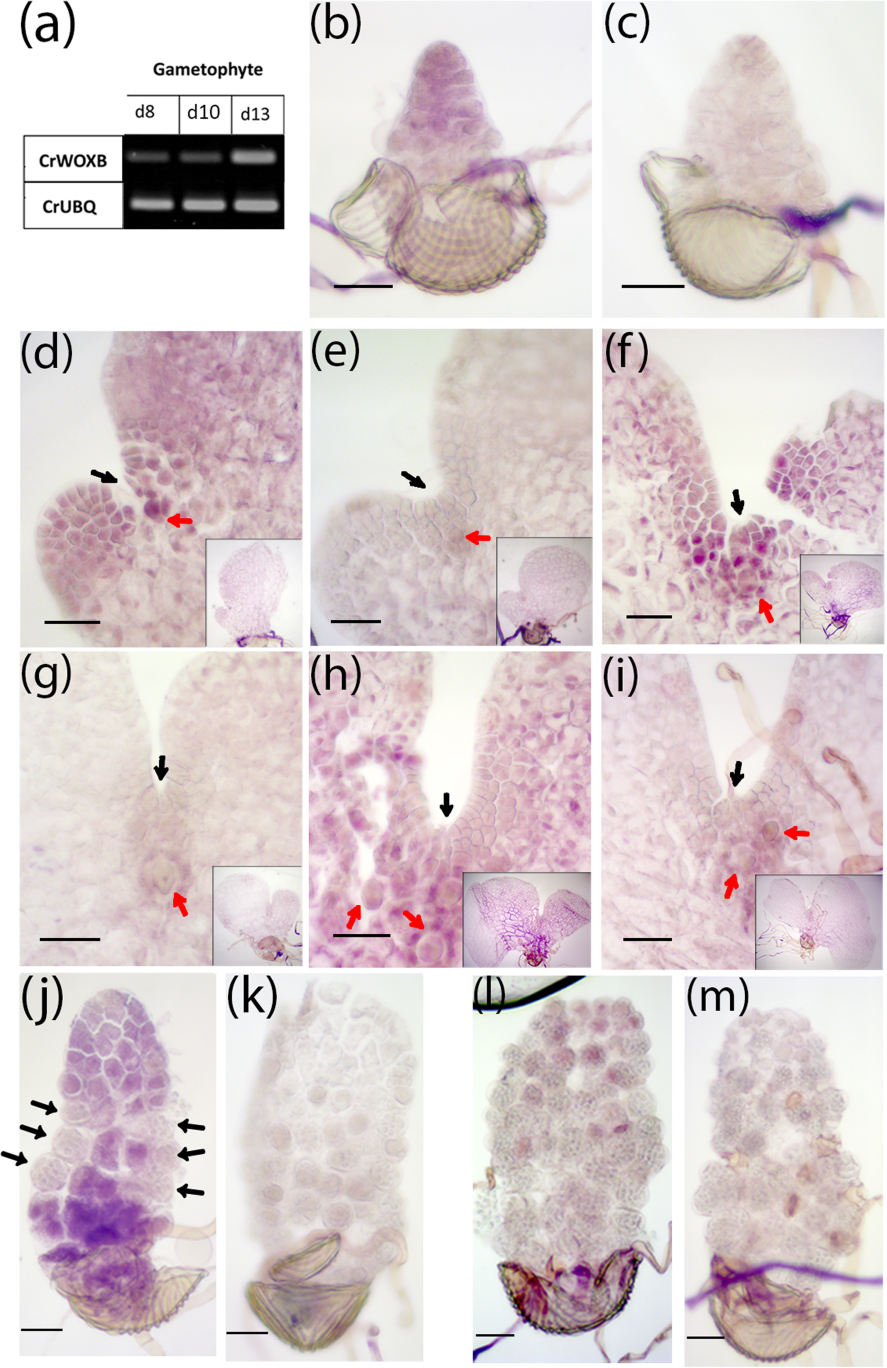
Expression of *CrWOXB* in the wild-type gametophyte localized to regions of cellular proliferation. **a** RT-PCR of *CrWOXB* expression in gametophytes; d8, d13, 8- and 13-days post-plating, respectively. *CrUBQ* used as control. (**b**-**m**) In-situ hybridization of whole-mount emerging (**b**, **c**), 8-day (**d**, **e**), 10-day (**f**, **g**) and 13-day (**h**, **i**) hermaphroditic gametophytes; (**c**, **e**, **g**, **i**) are sense controls, black and red arrows (**b**-**i**) indicate the notch-meristem regions and archegonia, respectively. Insets show staining in whole gametophytes. (**j**-**m**) In-situ hybridization of male gametophtyes before (**j**, **k**) and after (**l**, **m**) antheridium differentiation; (**k**, **m**) are sense controls, arrows in (**j**) depict differentiated antheridia. Scale bar = 0.05 mm

The expression of *CrWOXB* was observed in developing gametophytes and in sporophyte leaves. *CrWOXB* was expressed at low but discernable levels before (d8 and d10) sexual maturation and increased in gametophytes at sexual maturity (d13) (Fig. 1a). Whole-mount in situ hybridization revealed that *CrWOXB* mRNA was expressed in recently germinated gametophytes (Fig. 1b, c) and then in the notch meristem region in gametophytes before (d8, Fig. 1d, e; day 10, Fig. 1f, g) and after (d13, Fig. 1h, i) sexual maturation. Consistent with increased expression at d13 detected by RT-PCR analysis (Fig. 1a), d13 gametophytes showed a broader area of *CrWOXB* expression than d8 and d10, that was also further away from the notch region. In addition, *CrWOXB* expression was also detected in developing archegonia (Fig. 1d, f red arrows) suggesting a role in organ specification in hermaphrodites. At d8, similar to hermaphrodites, male gametophytes expressed *CrWOXB* in cells prior to antheridia development (Fig. 1j). The expression declined in d13, when most cells have developed into antheridia (Fig. 1l). Thus, the expression of *CrWOXB* in gametophytes is in actively dividing region in both hermapthrodites and males and the notch meristem and the archegonium of hermaphrodites.

**Fig. 2:**
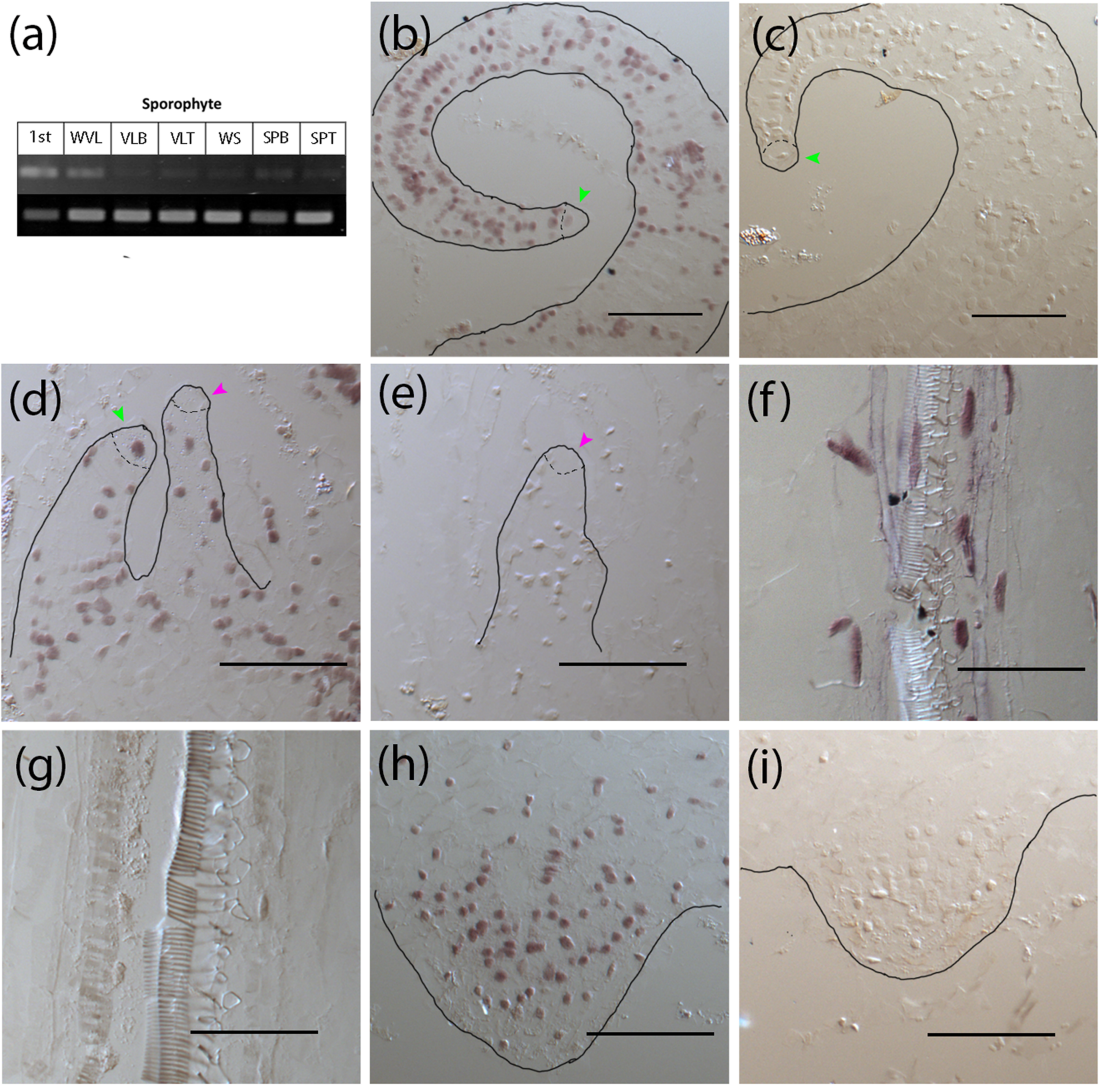
Expression of *CrWOXB* in the wild-type sporophyte localized to developing tissues and regions of cellular proliferation. **a** RT-PCR of *CrWOXB* expression in sporophytes; 1st, sporophytes with first leaf emerged; WVL, whole vegetative leaf; VLB, vegetative leaf pinna base; VLT, vegetative leaf pinna tip; WS whole sporophyll; SPB, sporophyll pinna base; SPT, sporophytll pinna tip. *CrUBQ* used as control. **b**-**i** In-situ hybridization of sectioned young sporophytes with 10-11vegetative leaves. Black outline depicts boundary of tissues; green arrowheads, leaf apical cell; pink arrowheads, shoot apical cell. **b**, **c** Emerging vegetative leaf primordia. **d**, **e** Shoot meristem and young leaf primordia. **f**, **g** Vascular bundles of older leaf tissues. **h**, **i** Root primordia. (**b**, **d**, **f**, **h**) anti-sense, (**c**, **e**, **g**, **i**) sense control. Scale bar = 0.1 mm (**b**-**i**)

The first set of leaves (total of 16–21 leaves) formed by *C. richardii* sporophytes are vegetative (do not produce sporangia), followed by an indeterminate number of sporophylls (produce sporangia). Pinnae refer to the leaflets of both vegetative and sporophyll leaves. The expression of *CrWOXB* in sporophyte tissues detected by RT-PCR showed the highest expression in sporophytes with one fully expanded leaf and in the later vegetative leaves but was barely detectable in the sporophylls, the pinna-base and tip of the vegetative leaves (Fig. 2a). *CrWOXB* mRNA was detected by in situ in young vegetative leaves (Fig. 2b), the shoot tip (Fig. 2d), leaf vascular bundles (Fig. 2f), and in root primordia (Fig. 2h) of young sporophytes bearing 10–11 vegetative leaves, when the first visible fiddlehead was observed. Notably *CrWOXB* expression is seen within the apical cells of leaf primordia and more mature leaf (Fig. 2b, d, green arrowhead) but not in the apical cell of the SAM (Fig. 2d, purple arrowhead). Thus, *CrWOXB* expression is localized to regions of actively dividing cells in the sporophyte generation.

### CrWOXB is required for proper growth of gametophytes

**Fig. 3:**
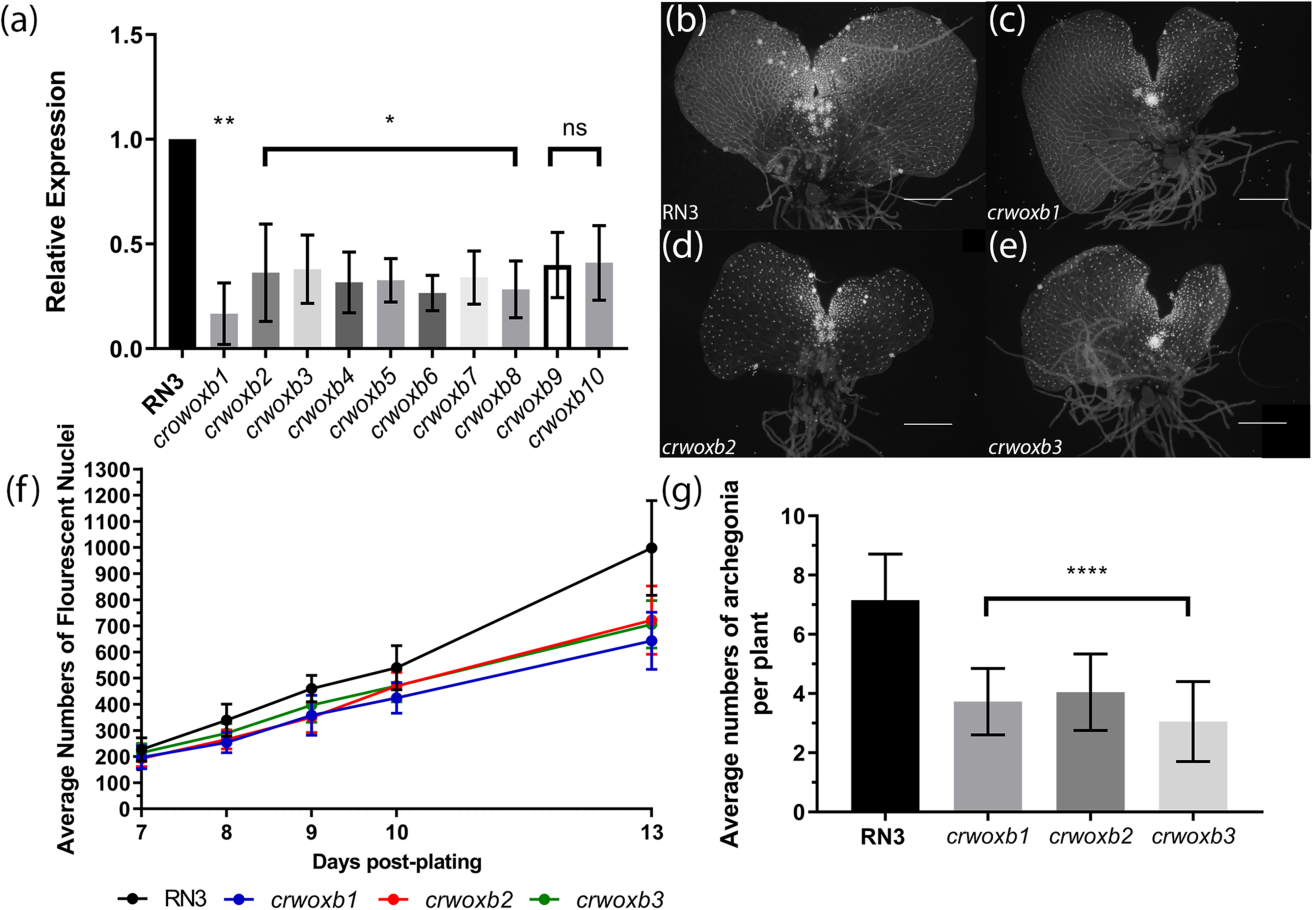
Reduced expression of *CrWOXB* decreases cell number of transgenic gametophytes. **a** Expression of *CrWOXB* in *CrWOXB* RNAi lines standardized to *CrUBQ* (*N* = 3), One-Way ANOVA (*, *p* < 0.05; ** *p* < 0.01 df = 4). **b**-**e** Fluorescent images of d13 gametophytes stained with Hoechst dye. **f** Average number of fluorescent nuclei of d7 to d13 old gametophytes (*N* ≥ 15). **g** Average numbers of archegonia present in d13 sexually mature gametophytes (*N* ≥ 18), One-Way ANOVA (****, *p* < 0.0001, df = 52). Scale bar = 0.5 mm. Error bars represent standard deviation

To carry out functional analyses, we created *CrWOXB* RNAi suppression, *crwoxb,* lines, by using *Agrobacterium*-mediated transformation of gametophytes. Suppression of *CrWOXB* transcripts were quantified in young sporophytes with 6–7 fully expanded vegetative leaves by RT-qPCR. *CrWOXB* expression in T_2_ sporophyte of *crwoxb* lines were found to express a range of levels from a high average of ~ 40% in *crwoxb10* to ~ 16% in *crwoxb1* compared to wild-type plants (Fig. 3a). The variation is likely due to position effect of the transgene [37]. No plants were recovered with undetectable *CrWOXB* expression.

Consistent with expression in the meristem and other regions of cell division (Fig. 1b-h), d13 gametophytes of *crwoxb* lines were smaller and had an altered morphology, including a wider notch (Fig. 3b-e; Additional file 2: Figure S2a-d inset) between the two lobes of the thallus. The wider notch appeared to be the result of a localized combination of fewer trapezoidal meristem cells and altered cell division planes, which prevented the lobes of the gametophyte from growing together. To quantify the gametophyte size, gametophyte nuclei were stained and counted. Wild-type and *crwoxb* lines produced similar numbers of gametophyte cells prior to d8. After d8, development of *crwoxb* lines is delayed by 1 day (Fig. 3f; Additional file 5: Table S2). The gametophyte notch meristem is generally formed from d7 to d8. The average numbers of cells produced by *crwoxb* gametophytes were less than wild-type gametophytes and the difference increased with time (Fig. 3f; Additional file 5: Table S2).

Archegonia house the eggs and are direct derivatives of the notch meristem in *C. richardii* [38]. Because the gametophytes of *crwoxb* lines had fewer cells, we hypothesized that they would also develop fewer archegonia. To test this, we compared numbers of archegonia in wild-type and the *crwoxb* lines and found that indeed the *crwoxb* lines produced fewer archegonia than wild-type plants (Fig. 3g; Additional file 6: Table S3). The archegonia of the *crwoxb* lines were functional as they produced sporophytes. The reduction in archegonia numbers could be due to fewer cells of *crwoxb* gametophytes or to involvement of CrWOXB in the specification of archegonia progenitor cells. To distinguish between these two possibilities, we compared the number of archegonia to number of cells in the entire gametophyte of wild-type and *crwoxb* lines (Additional file 6: Table S3). Thirteen-day-old *crwoxb* gametophytes, although having fewer cells, had on average 55 more, not fewer, cells for each archegonium than wild-type gametophytes. This result rules out the first but not the second scenario.

### CrWOXB promotes leaf development in the sporophyte generation

**Fig. 4:**
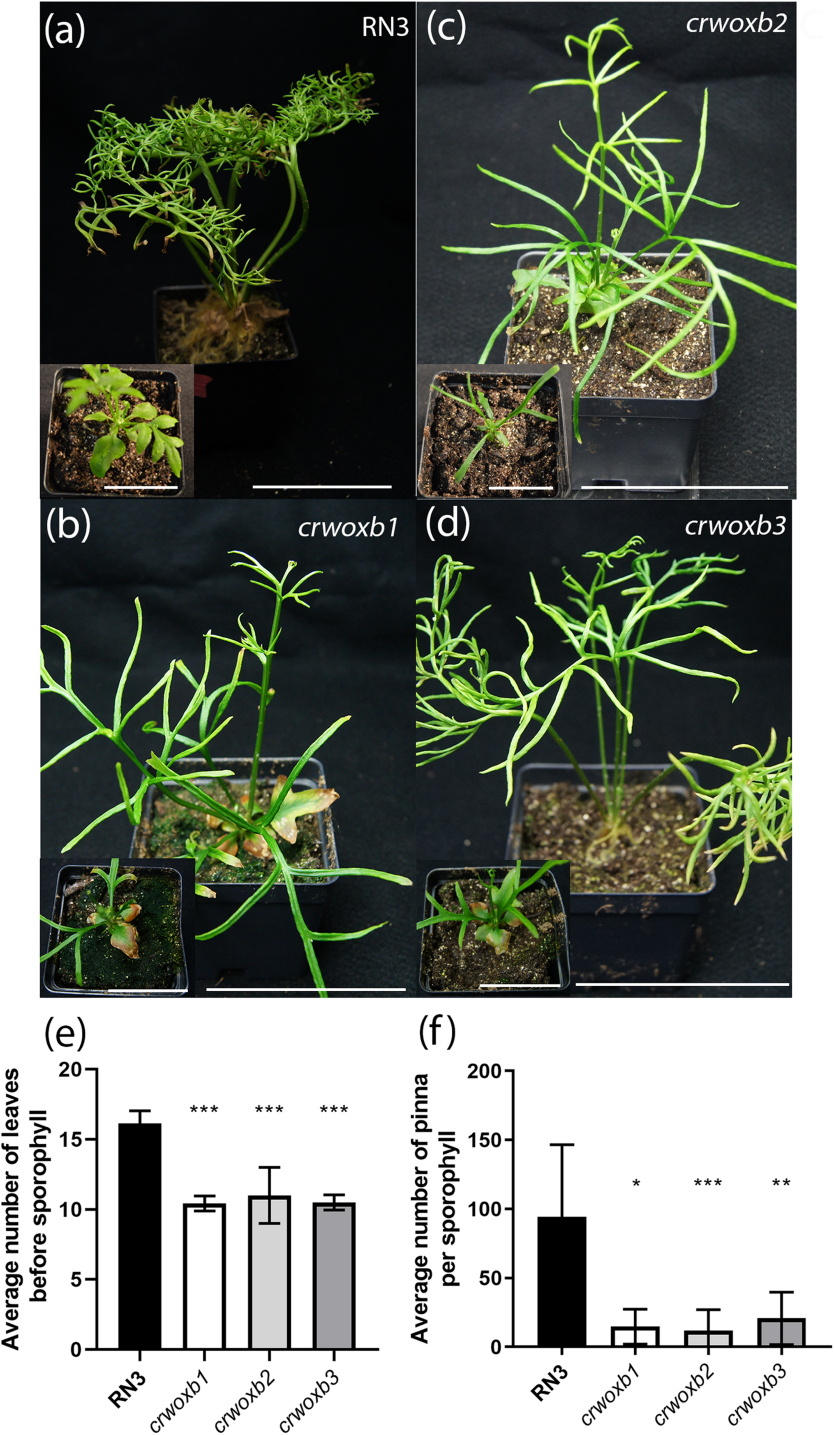
Transgenic sporophytes produce simpler leaves. **a**-**f** Sexually mature sporophytes producing sporophylls. RN3 are wild-type plants. (**a**-**d** inset) Young sporophytes of lines depicted in **a**-**d**. **e** Average numbers of leaves produced before the first sporophyll (*N* ≥ 6 plants), One-Way ANOVA (***, *p* < 0.001). **f** Average numbers of pinna per sporophyll (*N* ≥ 11 fronds). Scale bar = 9.3 cm. One-Way ANOVA (*, p < 0.05; **, p < 0.01; ***, p < 0.001 df = 24) (**a**-**d**). Scale bar = 4.65 cm (**a**-**d** inset). Error bars represent standard deviation

In situ hybridization revealed localized expression of *CrWOXB* in leaf primordia and developing leaves in the sporophyte (Fig. 2b, d), consistent with a role of leaf initiation. These results prompted an examination of leaf initiation and development in the *crwoxb* lines. In the wild type, 16–21 vegetative leaves are formed, followed by sporophylls (Fig. 4e). In contrast, *crwoxb* lines initially produced fewer vegetative leaves (Fig. 4e) before producing sporophylls. The number of sporophylls produced seemed to be comparable to the wild-type as both continue producing sporophylls indefinitely; however, the number of pinnae of each sporophyll was greatly reduced compared to that of wild-type plants (Fig. 4f). These results indicate a role of CrWOXB in leaf morphogenesis during both the vegetative and reproductive phases of the sporophyte generation.

### CrWOXB promotes root and lateral root initiation during sporophyte development

**Fig. 5:**
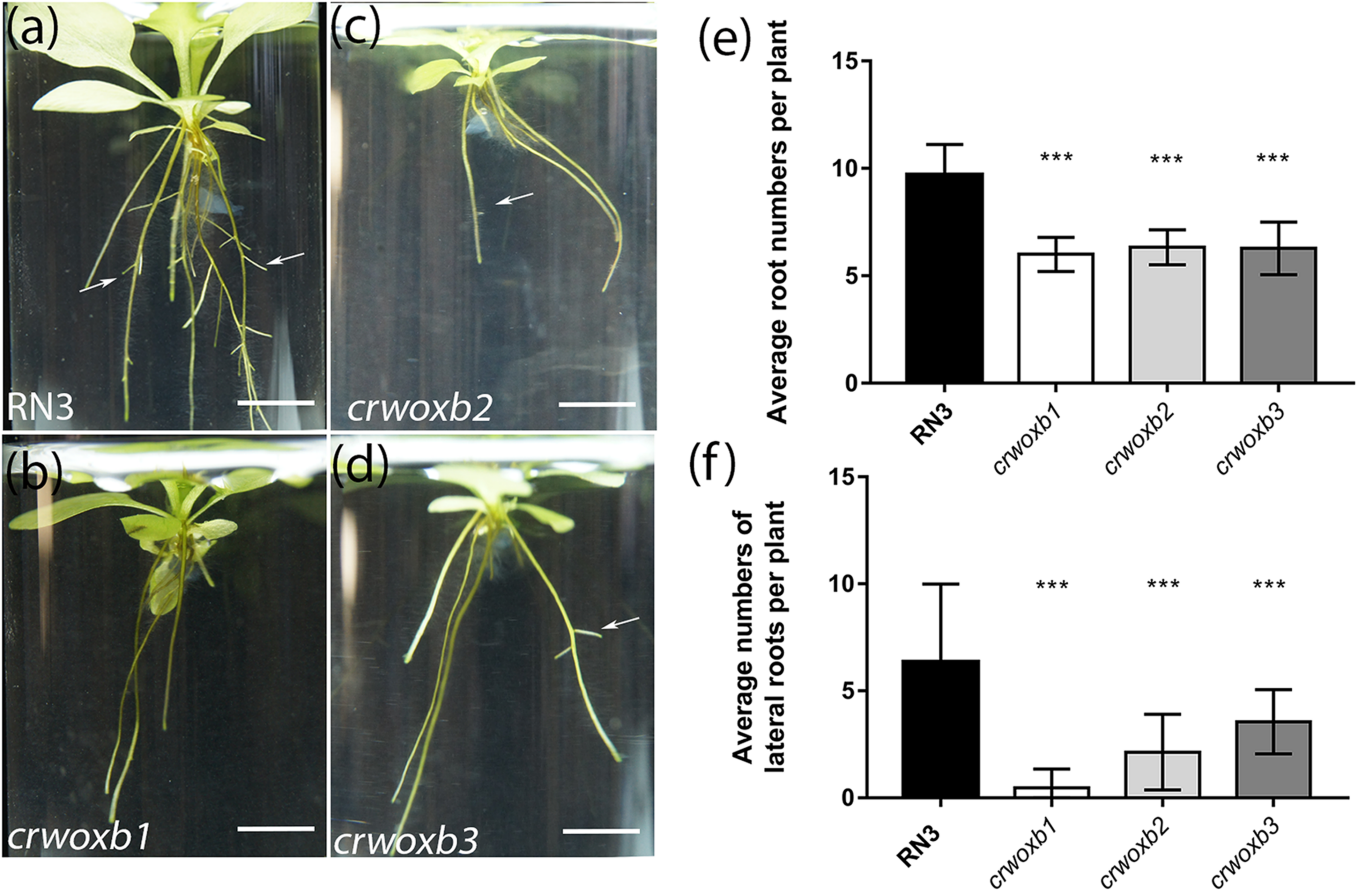
Transgenic sporophytes produce fewer roots and lateral roots. (**a**-**d** Images of lateral root growth from wild-type and transgenic plants grown for 2 weeks in liquid culture (white arrows show lateral roots). **e** Average number of roots per plant (*N* ≥ 14 sporophytes from each line). **f** Average numbers of lateral roots per plant (N ≥ 14 sporophytes from each line). **e**, **f** One-way ANOVA (***, p < 0.0001 df = 86). Scale bar = 8 mm. Error bars represent standard deviation

The expression of *CrWOXB* in root primordia (Fig. 2h) and during lateral root formation suggested a role for CrWOXB in root initiation and/or development. Sporophytes with 6–7 fully expanded leaves were grown in liquid media for 14 days before root observation. Wild-type sporophytes (Fig. 5a) exhibited more roots and were more branched than *crwoxb* lines (Fig. 5b-d). When quantified, the average number of roots (Fig. 5e) and lateral roots per plant (Fig. 5f) were significantly reduced in *crwoxb* lines compared with wild-type plants. Similar to leaf initiation and development, the significant reduction of root and lateral root numbers further confirmed the role of CrWOXB in organ initiation.

## Discussion

The *WOX* genes, especially the modern clade member *WUS*, are well studied in angiosperms. Considering their important roles in meristem maintenance, how these genes function in the ferns will help the understanding of fern meristems and their maintenance. Here we presented the first functional analysis of a fern *WOX* gene, *CrWOXB*, and showed its role in both gametophyte and sporophyte generations.

### *CrWOXB* functions in both the gametophyte and sporophyte generations

Reduced cell divisions in hermaphroditic gametophytes of *crwoxb* lines suggested that CrWOXB promotes cell division, mirroring intermediate WOX proteins in *A. thaliana* and *P. abies* where these proteins activate cyclin genes, which regulate the progression of the cell cycle [30, 39, 40]. In addition to CrWOXB’s function in cell division, in the hermaphrodites CrWOXB also seemed to play a role in specifying cells to become archegonia (Fig. 1g; Additional file 6: Table S3) in the region where *CrWOXB* is expressed highly. The reduced number of archegonia in *crwoxb* lines could be explained by non-cell autonomous action of CrWOXB, where decreased expression in *crwoxb* lines would need more cells to produce some threshold concentration for specification. Once specified, the egg-cell develop normally: its maturation and embryo development were unaffected in *crwoxb* lines, based on the observation that sporophytes formed after 5 days post-fertilization in both wild-type and *crwoxb* gametophytes. In the male, cell proliferation is followed closely by differentiation into antheridia [41, 42]. We detected *CrWOXB* in cells before, but not after differentiation into antheridia in d8 gametophytes (Fig. 1j, l).

In the sporophyte generation, abnormal phenotypes were observed in both the shoot and the root of *crwoxb* lines. In the shoot, the number of sterile fronds and pinna of the fertile fronds was diminished and similarly in the root, both root and lateral-root numbers were decreased. Therefore, we conclude that CrWOXB functions to promote cell division and possibly to specify organ formation in both generations of *C. richardii*.

*CrWOXB* functions in both generations, whereas its ortholog in *A. thaliana*, *AtWOX9,* has only been shown to function in sporophytes [27, 43]. Prior to this work, only the ancient clade of *WOX* genes have been shown to function in both the gametophyte and the sporophyte generations of *P. patens* [32]. The trend of diminishing *WOX* gene function in the gametophytes during evolution is consistent with the comparative transcriptome profiling between the moss *Furnaria hygrometrica* and *A. thaliana*, in which an enrichment of bryophyte gametophyte-biased transcription factors are found in sporophyte-biased (and sporophyte-specific) *A. thaliana* orthologs [44].

### The role of *CrWOXB* in the meristems of gametophytes and sporophyte

In the gametophyte, *CrWOXB* was expressed in both the male and hermaphrodite. Expression in the male persisted briefly, during thallus growth, before cells differentiate into antheridia. Similarly, in the hermaphrodite, *CrWOXB* was expressed shortly after spore germination; however, its expression appeared in the notch region, during and after the emergence of the lateral meristem. The expression pattern of *CrWOXB* in the male and hermaphrodite is in agreement with cell proliferation regions delineated by [42]. Our results suggest that *CrWOXB* function is required soon, if not immediately, after spore germination.

All growth in *C. richardii* sporophytes*,* as in other ferns, can be traced back to single, apical cells [45]. The shoot apical cell of *C. richardii* sits atop a slender stalk of meristem cells in a region defined as the proliferation zone [36]. Leaf initiation commences with the specification of one of the peripheral shoot meristem cells as a leaf apical cell, which persists throughout leaf development [46, 47]. Roots similarly are formed by the persistent action of a root apical cell [48]. Expression of *CrWOXB* is homogenous in the shoot, root, leaf primordia, and the vascular tissues signifying a more general role of CrWOXB in each region of cell proliferation. The homogenous expression pattern of *CrWOXB* in the primordia is similar to what was observed of *AtWOX9* in Arabidopsis shoot apical meristem [9, 40]. The expression pattern of *CrWOXB* in the root primordia is similar to that in the mature root tip reported by Nardmann et al. [11], and is expressed in tissues outside of where *CrWOXA*, the other intermediate *CrWOX* gene, is expressed, suggesting some functional divergence between these two paralogs. Interestingly, *CrWOXB* expression was not detected in apical cell of the shoot and root but was observed in the leaf primordia (Fig. 1h). We have consistently observed this difference, but the significance is unclear. The *CrWOXB* may be regulated differently in the shoot and root apices and in the leaf primordia.

It may not be surprising to find that *CrWOXB* played a role in both gametophytes and sporophylls. As observed by Hagemann [60], the pinnae of sporophyll and the gametophytes of the ferns are of structural similarity as both are dorsiventral with marginal meristematic growth and produce abaxial reproductive organs.

### The relationship between *CrWOXB* and the intermediate *WOX* transcription factor family

In *A. thaliana*, the homeodomain of *AtWOX8* and *AtWOX9* can partially rescue meristem function in a *wus-1* background, which establishes the intermediate WOX homeodomain as a key motif for meristem function [16]. Outside of the homeodomain, seed plant intermediate WOX members contain conserved N-terminal and C-terminal motifs that are not shared with intermediate clade proteins in *C. richardii*, CrWOXA and CrWOXB (Additional file 3: Figure S3). Despite the divergence outside the homeodomain, phenotypes of *crwoxb* in the shoot and root are reminiscent of *AtWOX9* null-mutant seedlings which fail to form leaves, secondary shoots and lateral roots [27]. The presence of the C-terminal domain and N-terminal motifs may be required for embryo patterning and development of the suspensor in *A. thaliana* because, in a complementary experiment, *AtWUS*, which does not contain the N- and C-terminal motifs of the intermediate clade, cannot rescue embryo arrest in *Atwox8 Atwox9* double mutants [16]. Therefore, in *C. richardii*, the homeodomain is the most likely motif involved in cell proliferation and organ specification in the gametophyte and sporophyte, while the divergent N- and C- terminal sequences may contain yet-to-be recognized motifs that have additional function during embryogenesis.

## Conclusion

We have functionally characterized an intermediate clade WOX protein CrWOXB throughout gametophyte and sporophyte development in the fern model *C. richardii* and found that *CrWOXB* is expressed in proliferating tissues of both generations. Knockdown *crwoxb* lines produce fewer gametophyte cells, and smaller sporophytes with fewer sporophyte organs, suggesting a conserved function in gametophytes and sporophytes despite their different architecture. Methods and results presented here serve as model for the analysis of the remaining *WOX* genes in *C. richardii* in order to understand how this gene family has diversified its functions in proliferative regions of the gametophyte and sporophyte generations.

## Methods

### Plant growth conditions

Spores of *C. richardii* strain Rn3 (wild-type) were originally obtained from Carolina Biological Supply Company (Burlington, NC). Wild-type and *CrWOXB* RNAi suppression lines (*crwoxb*) were surface sterilized in 4% sodium hypochlorite and 0.5% Tween-20 for 5 min, rinsed 4–5 times with sterile water and incubated at room temperature in the dark for 3–5 days to synchronize germination. Spores were then plated on basal media (1/2 MS, pH 6.0) supplemented with 100 μg ml^− 1^ ampicillin and maintained in humidity domes at 26 °C with a light/dark cycle of 16/8 under light intensity of 100 μM m^− 2^ s^− 1^ for gametophyte development. Plates were inverted after 10 days of growth (d10) to deter fertilization. Sporophytes were grown in BLP germination soil #1 (Beautiful Land Products, West Branch, IA) under humidity domes in the same light and temperature regime as gametophytes.

### Transformation of *C. richardii* gametophytes

A 302-bp fragment (See Additional file 4: Table S1 for primer sequences) of *CrWOXB* was cloned into vectors pK7GWIWG2 and pH7GWIWG2 to generate *CrWOXB* RNAi constructs using the Gateway technology as described by Curtis and Grossniklaus [49] and Bui et al. [50]. Each construct was introduced into *Agrobacterium tumefaciens* strain GV3101 from *Escherichia coli* with an *E.coli* helper strain containing the pRK 2013 plasmid [51]. Stable transformation of young gametophyte tissue was conducted as described previously [52]. Successfully transformed gametophytes (T_0_) were selected on media containing 50 μg ml^− 1^ kanamycin or 5 μg ml^− 1^ hygromycin. Resistant gametophytes were isolated and allowed to self-fertilize to produce sporophytes (T_1_). Sporophytes were moved to liquid basal media and allowed to root before transplanting to soil. From the more than 20 independent transgenic lines isolated, 10 were chosen for qPCR analysis and characterization. Detailed phenotyping of three lines are presented here.

### Whole-mount and sectioned in situ hybridization

Antisense and sense RNA probes used for in situ hybridization experiments were synthesized from 1 μg of PCR products amplified using primers containing T7 promoter sequences (Additional file 4: Table S1) with T7 RNA polymerases (Agilent, Santa Clara, CA), and DIG RNA labeling mix (Roche Diagnostics, Indianapolis, IN). DIG-labeled RNA probes were precipitated in 2.25 M LiCl overnight at − 20 °C, before resuspension in nuclease-free water. RNA concentration was measured with a Nanodrop One (Thermo-Scientific, Waltham, MA) and then diluted 1:1 with deionized formamide and stored at − 20 °C.

The SAM from young sporophytes with 10–11 vegetative leaves, the youngest a visible fiddlehead, was dissected and vacuum infiltrated with fixing solution (4% paraformaldehyde in 1x PBS) for 45 min and then incubated in fixing solution overnight at 4 °C. Dehydration, embedding, pre-hybridization, hybridization and post-hybridization washes were based on Jackson [59], except that acetic anhydride washes were omitted from pre-hybridization. Embedded tissues were sectioned at 8 μm thickness with a rotary microtome. Probe detection and color development protocols were based on Ambrose et al. [58]. Whole-mount in situ was adapted from the protocol of Ambrose et al. [36, 58], with the following modifications. Gametophytes were fixed in FAA (formaldehyde: ethanol: acetic acid, 3.7%:50%:5% v/v respectively) at room temperature for 1 h, then stored in 70% ethanol at − 20 °C. Fixed gametophytes were processed without Histoclear II. Color development of whole-mount in situ tissues was stopped in ddH_2_O and mounted in 50% glycerol. Whole-mount samples were viewed with a Zeiss compound light microscope and imaged with the Zeiss Axiocam ERc 5 s digital camera (Carl Zeiss Microscopy LLC, Thornwood, NY). DIC images of sectioned samples were viewed with a Nikon Eclipse E800 (Nikon Instruments Inc., Melville, NY) and captured with a Photometrics CoolSNAP cf. (Photometrics, Tucson, AZ). To confirm gene expression patterns, each in-situ experiment was repeated at least two times using different biological samples.

### RNA extraction and RT-PCR analyses

Gametophyte and sporophyte tissue were harvested and flash frozen in liquid nitrogen, then stored at − 70 °C. Total RNA was extracted from frozen tissue with the Quick-RNA MiniPrep (Plus) kit (Zymo Research, Irvine, CA) and 750 ng of gametophyte total RNA or 500 ng of sporophyte total RNA was used in reverse transcriptase reaction using MMLV (New England Biolabs, Ipswich, MA) with N9 random primers (IDT Coralville, IA). PCR was conducted with the following cycles: 2 min at 94 °C, followed by 37 cycles of 30s at 94 °C, 30s at 59 °C, and 30s at 72 °C, with a 5 min final extension time at 72 °C for *CrWOXB*, and 25 cycles under the same conditions for *CrUBQ* transcripts.

For RT-qPCR, three biological and two technical replicates were performed for each line. Total RNA from whole young sporophytes with 6–7 fully expanded round leaves was extracted and 200 ng were used in cDNA synthesis as described above. Due to delay in development of *crwoxb* lines, the age of the sporophytes in both the wild-type and *crwoxb* lines was determined by numbers of leaves and not days. Primers for qPCR are listed in Additional file 4: Table S1. Detection of amplification was performed using SYBR green chemistry (Roche Diagnostic, Indianapolis, IN) with the Roche LightCycler 480 Real-Time PCR system (Roche Diagnostic). The PCR cycle was as follows: 10 min at 95 °C, followed by 45–55 cycles of 10s at 95 °C, 10s at 62 °C, and 20s at 72 °C, with a single fluorescence read at the end of each extension time. A melting curve analysis was also performed and analyzed using the Tm calling software module to verify the absence of primer dimers and non-specific products. Calibrator normalized relative quantification was performed using the 2nd derivative maximum algorithm with three internal relative standards. *CrWOXB* expression was measured relative to *CrUBQ*.

### Phenotypic analysis of *crwoxb* lines

To count cells of the gametophytes, gametophytes were cleared overnight in 100% ethanol at 4 °C, then rinsed 3 times for 5 min in water and stained with Hoechst 33342 (40 μg ml^− 1^) (Invitrogen, Carlsbad, CA) for at least 15 min, rinsed in water and mounted on slides with 50% glycerol. Gametophytes were then imaged with a Leica stereomicroscope and a Qicam camera (Qimaging, Surrey, BC, Canada) with a DAPI filter. Nuclei of gametophytes were counted in Photoshop CC (Adobe systems, San Jose, CA). Brightness and contrast were increased slightly to facilitate cell counting.

For root and lateral root counts, spores of wild-type and *crwoxb* lines were grown on basal media for 13 days, after which individual hermaphroditic gametophytes were isolated for self-fertilization by adding a few drops of water. Resulting sporophytes were transferred to 100 ml of liquid basal media with 100 μg ml^− 1^ ampicillin and grown for an additional 2 weeks before roots and lateral roots were counted. Vegetative leaves and pinnae were counted on soil-grown sporophytes when each sporophyte had 5–7 sporophylls.

### Statistical evaluation of the data

Statistical analyses of *CrWOXB* levels in *crwoxb* lines, gametophyte archegonia numbers, and sporophyte phenotypes were conducted with one-way ANOVA, while gametophyte cell numbers were conducted with two-way ANOVA. Both analyses were followed by Dunnett’s multiple comparisons test. All calculations were done in GraphPad Prism version 8.0.1 (GraphPad Software, San Diego, CA).

### Phylogeny of WOX proteins

Multiple sequence alignments of WOX homeodomains are based on T-Coffee [53] and trees were built using the Maximum-Likelihood method in phyML [54] with 500 bootstrap replicates and visualized in MEGA7 [55]. Protein sequences for *Ostreococcus tauri*, *Osctreococcus lucimarinus*, *Physcomitrella patens*, *Selaginella kraussiana, Oryza sativa* were obtained from Phytozome [56]. *Azolla filiculoides*, *Salvinia cuculata* sequences were obtained from Fernbase [57]. *Ceratopteris richardii* sequences were obtained from NCBI. *Arabidopsis thaliana* sequence were obtained from TAIR. Full length protein sequences are provided in Additional file 7.

## Additional files

Additional file 1: **Figure S1.** Phylogeny of WOX proteins from representative embryophytes. Abbreviations Ot, *Ostreococcus tauri*; Ol, *Osctreococcus lucimarinus*; Pp, *Physcomitrella patens*; Sk, *Selaginella kraussiana*; Af, *Azolla filiculoides*; Sc, *Salvinia cuculata*; Cr, *Ceratopteris richardii*; Os, *Oryza sativa*; At, *Arabidopsis thaliana*. Evolutionary history was inferred using Maximum-likelihood method with 500 bootstrap replicates as a test of relatedness. Alignment of sequences was conducted with T-Coffee and cladogram constructed in phyML and visualized with MEGA7. The WOX genes in the two sequenced fern genomes, *Azolla* and *Salvinia* [58] have not been previously included in constructing any WOX tree. Asterisk denote outgroup. (DOCX 1484 kb)

Additional file 2: **Figure S2.** Bright-field microscopy of d13 transgenic gametophytes. (a) RN3 (wild type). (b-d) transgenic gametophytes. (a-d inset) 2x digital-enlargement of a ~ 250 × 250 pixel region of the notch meristem. Red lines outline the notch meristem, in (a) the prothallus overlaps and no space is present between lobes of the prothallus. Scale bar = 0.5 mm. (DOCX 12696 kb)

Additional file 3: **Figure S3.** Protein domain comparison of intermediate clade WOX proteins from *A. thaliana* and *C. richardii*. (a) Model of intermediate clade WOX proteins from *C. richardii* and *A. thaliana*, green boxes represent the homeodomain and blue the C-terminal domain. (b) T-Coffee sequence alignment of *C. richardii* and *A. thaliana* intermediate WOX proteins domains. Specific motifs are outlined, and conserved residues are highlighted in green, less conserved sites are highlighted in yellow. (DOCX 160 kb)

Additional file 4: **Table S1.** Primer sequences used in the study. Sequences left of * are added for directional cloning or T7 promoter sequence. All sequences are 5′ to 3′. (DOCX 20 kb)

Additional file 5: **Table S2.** Average number of gametophyte cells observed at days 7 to 10 of growth and the average number of cells produced per day. *, *p* < 0.05; **, *p* < 0.01; ***, *p* < 0.001; ****, *p* < 0.0001, Two-way ANOVA. (DOCX 20 kb)

Additional file 6: **Table S3.** Transgenic and wild-type gametophyte cell and archegonia numbers at d13. ****, *p* < 0.0001. Two-way ANOVA, cell numbers or One-way ANOVA, archegonium numbers. (DOCX 20 kb)

Additional file 7: Sequences used in Phylogeny. (TXT 20 kb)

## Data Availability

See “Phylogeny of WOX proteins” section for the datasets used and/or analyzed during the current study.

## Abbreviations

CZ: Central zone
DAPI: 4′,6-diamidino-2-phenylindole
DIG: Digoxygenin
FAA: Formaldehyde: ethanol: acetic acid
mRNA: Messenger RNA
MS: Murashige and Skoog
NCBI: National Center for Biotechnology Information
OC: Organizing center
PBS: Phosphate buffered saline
RAM: Root apical meristem
RNAi: RNA interference
RT-PCR: Reverse transcription polymerase chain reaction
RT-qPCR or qPCR: Reverse transcription - quantitative polymerase chain reaction
SAM: Shoot apical meristem
TAIR: The Arabidopsis Information Resource
UBQ: Ubiquitin
WOX: *WUSCHEL-related homeobox*
WUS: *WUSCHEL*
