# Supplementary material for: A fern WUSCHEL-RELATED HOMEOBOX gene functions in both gametophyte and sporophyte generations
Source: BMC Plant Biol. 2019 Oct 11;19:416. doi: 10.1186/s12870-019-1991-8 (PMC6788082; doi:10.1186/s12870-019-1991-8)
Supplement: Supplementary file 1 — Figure S1. Phylogeny of WOX proteins from representative embryophytes. Abbreviations Ot, Ostreococcus tauri; Ol, Osctreococcus lucimarinus; Pp, Physcomitrella patens; Sk, Selaginella kraussiana; Af, Azolla filiculoides; Sc, Salvinia cuculata; Cr, Ceratopteris richardii; Os, Oryza sativa; At, Arabidopsis thaliana. Evolutionary history was inferred using Maximum-likelihood method with 500 bootstrap replicates as a test of relatedness. Alignment of sequences was conducted with T-Coffee and cladogram constructed in phyML and visualized with MEGA7. The WOX genes in the two sequenced fern genomes, Azolla and Salvinia [58] have not been previously included in constructing any WOX tree. Asterisk denote outgroup. (DOCX 1484 kb) [file 12870_2019_1991_MOESM1_ESM.docx]

**Figure S1.** Phylogeny of WOX proteins from representative embryophytes. Abbreviations Ot, *Ostreococcus tauri*; Ol, *Osctreococcus lucimarinus*; Pp, *Physcomitrella patens*; Sk, *Selaginella kraussiana*; Af, *Azolla filiculoides*; Sc, *Salvinia cuculata*; Cr, *Ceratopteris richardii*; Os, *Oryza sativa*; At, *Arabidopsis thaliana*. Evolutionary history was inferred using Maximum-likelihood method with 500 bootstrap replicates as a test of relatedness. Alignment of sequences was conducted with T-Coffee and cladogram constructed in phyML and visualized with MEGA7. The WOX genes in the two sequenced fern genomes, *Azolla* and *Salvinia* (54) have not been previously included in constructing any WOX tree. Asterisk denote outgroup.





Ancient

Intermediate

Modern
