# Supplementary material for: A fern WUSCHEL-RELATED HOMEOBOX gene functions in both gametophyte and sporophyte generations
Source: BMC Plant Biol. 2019 Oct 11;19:416. doi: 10.1186/s12870-019-1991-8 (PMC6788082; doi:10.1186/s12870-019-1991-8)
Supplement: Supplementary file 2 — Figure S2. Bright-field microscopy of d13 transgenic gametophytes. (a) RN3 (wild type). (b-d) transgenic gametophytes. (a-d inset) 2x digital-enlargement of a ~ 250 × 250 pixel region of the notch meristem. Red lines outline the notch meristem, in (a) the prothallus overlaps and no space is present between lobes of the prothallus. Scale bar = 0.5 mm. (DOCX 12696 kb) [file 12870_2019_1991_MOESM2_ESM.docx]

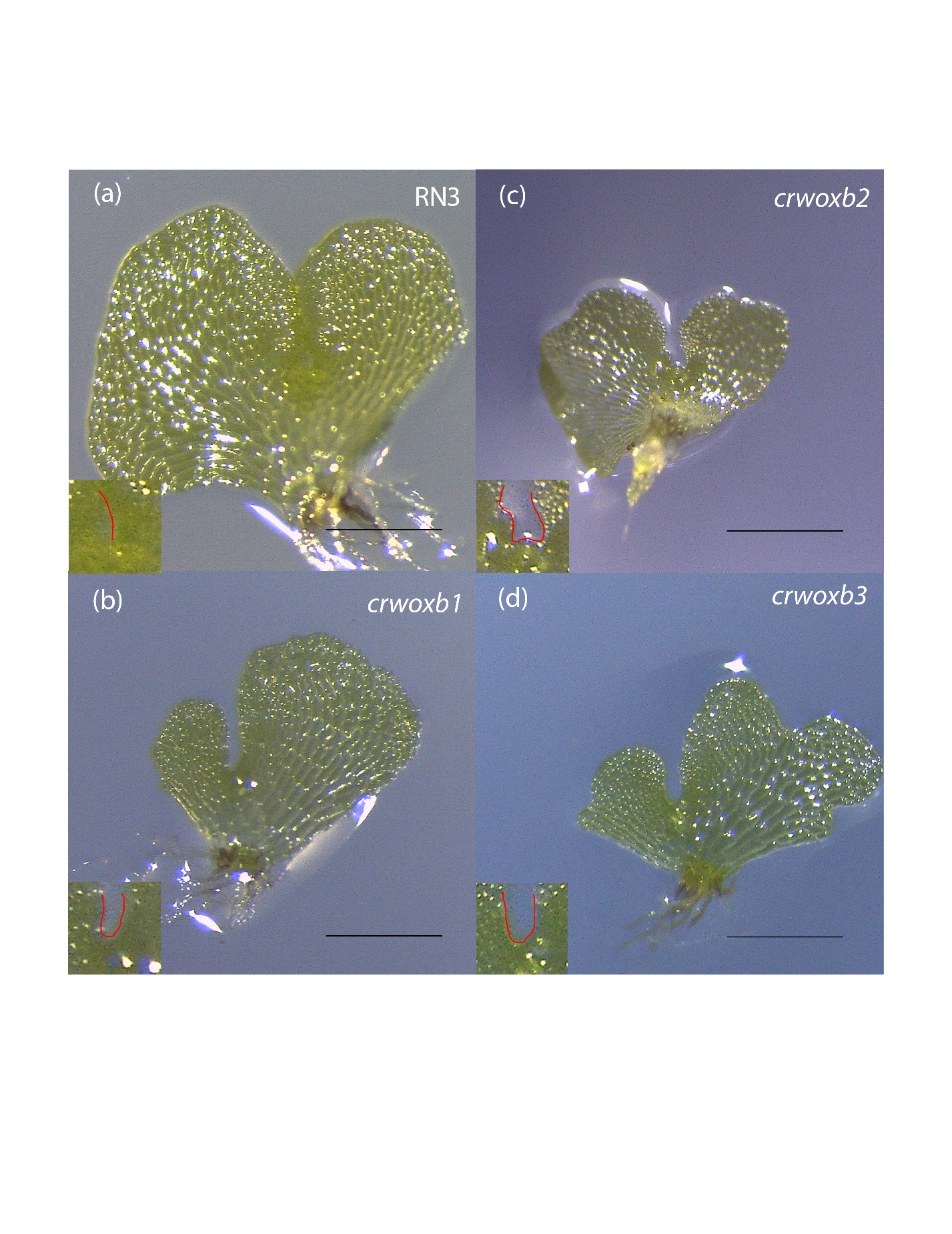


**Figure S2.**  Bright-field microscopy of d13 transgenic gametophytes. (a) RN3 (wild-type). (b-d) transgenic gametophytes. (a-d inset) 2x digital-enlargement of a ~250x250 pixel region of the notch meristem. Red lines outline the notch meristem, in (a) the prothallus overlaps and no space is present between lobes of the prothallus. Scale bar = 0.5mm
