# Supplementary material for: A fern WUSCHEL-RELATED HOMEOBOX gene functions in both gametophyte and sporophyte generations
Source: BMC Plant Biol. 2019 Oct 11;19:416. doi: 10.1186/s12870-019-1991-8 (PMC6788082; doi:10.1186/s12870-019-1991-8)
Supplement: Supplementary file 3 — Figure S3. Protein domain comparison of intermediate clade WOX proteins from A. thaliana and C. richardii. (a) Model of intermediate clade WOX proteins from C. richardii and A. thaliana, green boxes represent the homeodomain and blue the C-terminal domain. (b) T-Coffee sequence alignment of C. richardii and A. thaliana intermediate WOX proteins domains. Specific motifs are outlined, and conserved residues are highlighted in green, less conserved sites are highlighted in yellow. (DOCX 160 kb) [file 12870_2019_1991_MOESM3_ESM.docx]

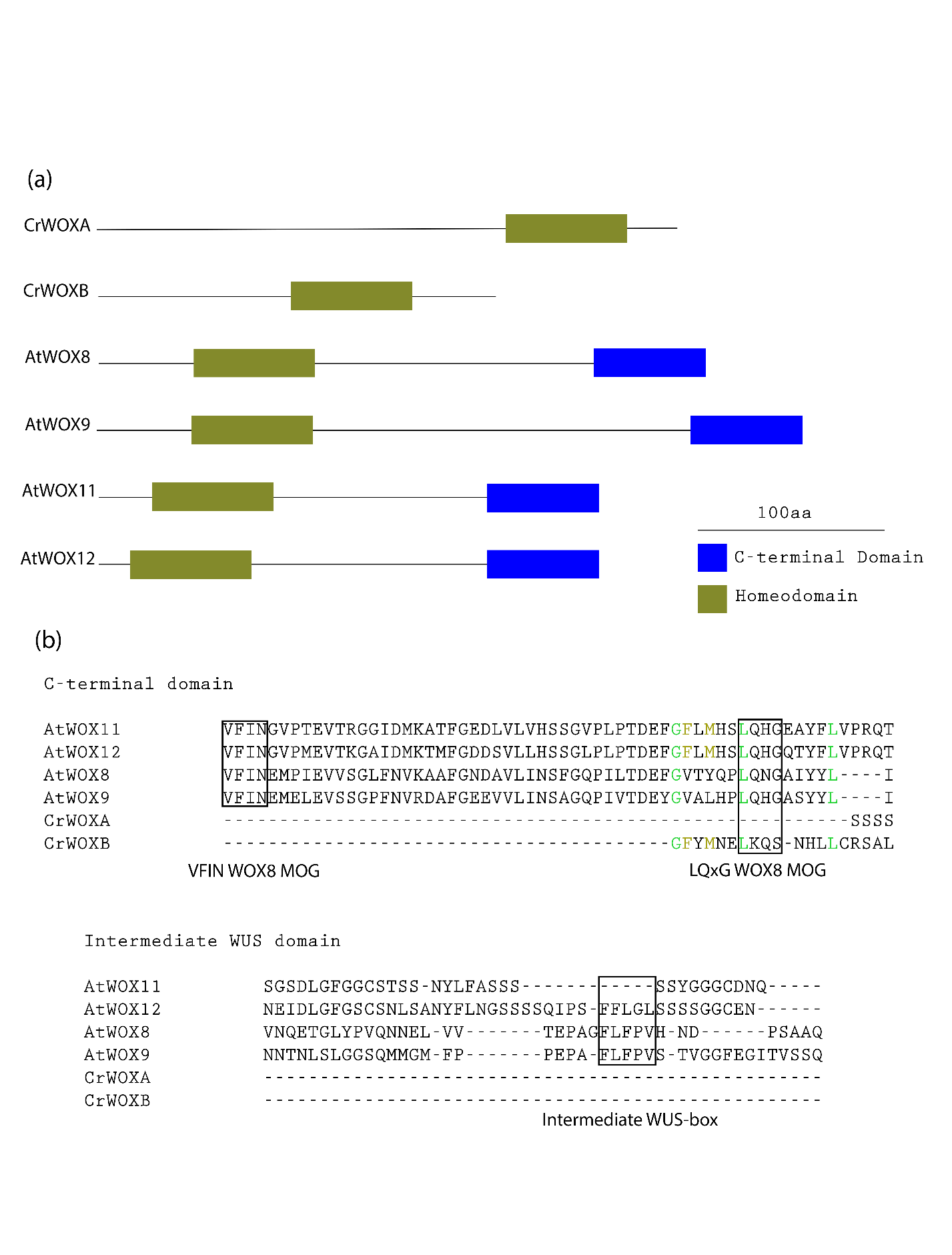


**Figure S3.** Protein domain comparison of intermediate clade WOX proteins from *A. thaliana* and *C. richardii*. (a) Model of intermediate clade WOX proteins from *C. richardii* and *A. thaliana*, green boxes represent the homeodomain and blue the C-terminal domain. (b) T-Coffee sequence alignment of *C. richardii* and *A. thaliana* intermediate WOX proteins domains. Specific motifs are outlined, and conserved residues are highlighted in green, less conserved sites are highlighted in yellow.
