# Supplementary material for: A fern WUSCHEL-RELATED HOMEOBOX gene functions in both gametophyte and sporophyte generations
Source: BMC Plant Biol. 2019 Oct 11;19:416. doi: 10.1186/s12870-019-1991-8 (PMC6788082; doi:10.1186/s12870-019-1991-8)
Supplement: Supplementary file 4 — Table S1. Primer sequences used in the study. Sequences left of * are added for directional cloning or T7 promoter sequence. All sequences are 5′ to 3′. (DOCX 20 kb) [file 12870_2019_1991_MOESM4_ESM.docx]

**Table S1.** Primer sequences used in the study.

| **Gene** | **Sequence** | **Use (Product length in bp)** |
| --- | --- | --- |
| *CrWOXB* | F-cacc* atggtattccatctcgctttcg | Insert for RNAi (302) |
|  | R-gtgttggccgttccatggc |  |
|  | F-cagcggtgcttgcacgc | Expression analysis RT-PCR, RT-qPCR (283) |
|  | R-agccattcgtaggagacgaaga |  |
|  | F-tggtattccatctcgctttcg | Anti-sense In-situ probe synthesis (302) |
|  | R-taatacgactcactatagggctgcc*gaactgattcagaca |  |
|  | F-taatacgactcactatagggtcggc*agcattgtagaag | Sense In-situ probe synthesis (200) |
|  | R-ttataaagcagaacggcatagtag |  |
|  |  |  |
| *CrUBQ* | F-gatggccgtactcttgcagac | Expression analysis RT-PCR, RT-qPCR (348) |
|  | R-ggagacgaagcacgagatga |  |

Sequences left of * are added for directional cloning or T7 promoter sequence. All sequences are 5’ to 3’
