# Supplementary material for: A fern WUSCHEL-RELATED HOMEOBOX gene functions in both gametophyte and sporophyte generations
Source: BMC Plant Biol. 2019 Oct 11;19:416. doi: 10.1186/s12870-019-1991-8 (PMC6788082; doi:10.1186/s12870-019-1991-8)
Supplement: Supplementary file 5 — Table S2. Average number of gametophyte cells observed at days 7 to 10 of growth and the average number of cells produced per day. *, p < 0.05; **, p < 0.01; ***, p < 0.001; ****, p < 0.0001, Two-way ANOVA. (DOCX 20 kb) [file 12870_2019_1991_MOESM5_ESM.docx]

**Table S2.** Average number of gametophyte cells observed at day 7 to 10 of growth and the average number of cells produced per day.

| **Day** | **Average number of cells per line, N, SD** | | | |
| --- | --- | --- | --- | --- |
|  | **RN3** | ***crwoxb1*** | ***crwoxb2*** | ***crwoxb3*** |
| 7 | 227.5, 21, 44.8 | 197.6, 15, 43.8 | 193.2, 15, 30.9 | 215.2, 20, 33.8 |
| 8 | 339.7, 22, 61.4 | 254.6^**^, 23, 39.7 | 265.8^*^, 20, 36.7 | 289.5, 20, 36.6 |
| 9 | 460.4, 20, 50.3 | 357.9^***^, 19, 76.7 | 350.2^****^, 20, 57.5 | 397^*^, 20, 66.3 |
| 10 | 540.1, 21, 84.7 | 424.8^****^,19, 58.6 | 469.9^*^, 20, 52.9 | 471.3^*^, 20, 61.7 |
| Delta = (d10-d7) | 312.6 | 227.2 | 276.7 | 256.1 |
| Growth of gametophytes (cells/day) = Delta/3 days of growth | 104.2 | 75.7 | 92.3 | 85.4 |
| Proportion of wild-type growth |  | 0.72 | 0.88 | 0.82 |

*, p <0.05; **, p <0.01; ***, p <0.001; ****, p <0.0001, Two-way ANOVA.
