# Supplementary material for: A fern WUSCHEL-RELATED HOMEOBOX gene functions in both gametophyte and sporophyte generations
Source: BMC Plant Biol. 2019 Oct 11;19:416. doi: 10.1186/s12870-019-1991-8 (PMC6788082; doi:10.1186/s12870-019-1991-8)
Supplement: Supplementary file 6 — Table S3. Transgenic and wild-type gametophyte cell and archegonia numbers at d13. ****, p < 0.0001. Two-way ANOVA, cell numbers or One-way ANOVA, archegonium numbers. (DOCX 20 kb) [file 12870_2019_1991_MOESM6_ESM.docx]

**Table S3.** Transgenic and wild-type gametophyte cell and archegonia numbers at d13.

|  | **RN3** | ***crwoxb1*** | ***crwoxb2*** | ***crwoxb3*** |
| --- | --- | --- | --- | --- |
| Avg. cells per line at d13 | 998.6 | 642.9^****^ | 722^****^ | 706.1^****^ |
| Number of gametophytes, Std. Deviation | 20, 181 | 22, 110 | 21, 131 | 21, 91 |
|  |  |  |  |  |
| Avg. number of archegonia per line at d13 | 7.2 | 3.7^****^ | 4.0^****^ | 3.1^****^ |
| Number of gametophytes counted, Std. Deviation | 20, 1.6 | 22, 1.1 | 22, 1.3 | 18, 1.3 |
| Number of cells produced/archegonia produced | 138.7 | 173.8 | 180.5 | 227.8 |

****, p < 0.0001. Two-way ANOVA, cell numbers or One-way ANOVA, archegonium numbers.
